# Did Maryland’s All-Payer Global Budget Revenue Model Change Hospital Margins and Revenue per Discharge? A Difference-in-Differences Analysis of Maryland and Massachusetts, 2010–2023

**DOI:** 10.1177/11786329261486708

**Published:** 2026-09-04

**Authors:** Kola Adegoke

**Affiliations:** 1 Department of Health & Biomedical Sciences, College of Health Professions, University of Texas Rio Grande Valley, Edinburg, TX, USA; 2 School of Health Sciences and Practice, 52146 New York Medical College, Valhalla, NY, USA

**Keywords:** Maryland all-payer model, global budget revenue, hospital finance, total margin, revenue per discharge, difference-in-differences, medicare, COVID-19

## Abstract

**Background:**

Maryland implemented an all-payer hospital global budget revenue (GBR) model in 2014 to decouple hospital revenue from volume and improve revenue predictability. This study evaluated whether Maryland’s 2014 GBR implementation was associated with differential changes in hospital total margins, inpatient revenue per discharge, and COVID-period margin volatility relative to Massachusetts.

**Methods:**

A matched hospital-year panel for Maryland and Massachusetts spanning 2010–2023 (2,261 matched observations) was constructed by merging Medicare cost report-based financial measures with provider characteristics using CMS certification identifiers. Two-way fixed-effects difference-in-differences models with hospital and year fixed effects and hospital-clustered standard errors estimated post-2014 changes in outcomes in Maryland relative to Massachusetts. Identification was assessed using an event-study specification and a placebo test (fake treatment in 2012, restricted to the pre-period). Financial stability under pandemic shock was evaluated using a COVID-period differential effect on margin volatility, defined as the absolute year-over-year change in total margin. An additional restricted sensitivity analysis for 2010–2019 adjusted for available case-mix and payer-mix covariates.

**Results:**

GBR implementation was not associated with a statistically detectable change in total margins (β = 0.021, p = 0.232). In contrast, GBR was associated with higher log inpatient revenue per discharge in Maryland relative to Massachusetts after 2014 (β = 0.287, p = 0.003), consistent with an approximate 33% increase in revenue per discharge (exp[0.287]−1). However, in the restricted 2010–2019 sensitivity analysis adjusting for available case-mix and payer-mix covariates, this association was attenuated and no longer statistically significant (β = 0.030, p = 0.513). Margin volatility during COVID-19 did not differ significantly between states (β = −0.018, p = 0.161). The placebo test showed no spurious effect in the pre-period (β = −0.008, p = 0.801), supporting the validity of the design.

**Conclusions:**

Maryland’s all-payer global budgets were associated with higher revenue per discharge in the primary model, but with no detectable change in total margins or in differential margin volatility during COVID-19. The revenue-per-discharge finding was sensitive to adjustment for available case-mix and payer-mix covariates in a restricted sensitivity analysis, supporting a cautious interpretation. These findings are consistent with GBR altering revenue intensity without clear evidence of improved profitability or shock-absorbing effects over 2010–2023.

## Plain Language Summary

Most U.S. hospitals operate under a fee-for-service payment model, in which increased service delivery results in higher revenue. This structure can elevate costs and contribute to revenue instability. In contrast, since 2014, Maryland has implemented global budgets, establishing a fixed annual hospital revenue target across all payers.

This study compared hospitals in Maryland and Massachusetts from 2010 to 2023 using Medicare cost-report financial data. The analysis focused on hospital profit margins, revenue per inpatient discharge (as a proxy for revenue intensity), and the extent of margin fluctuations during the COVID-19 period.

Following the adoption of global budgets in Maryland, hospital revenue per discharge increased more in Maryland than in Massachusetts, while hospital margins did not change statistically. However, this revenue-per-discharge finding was less robust in a restricted sensitivity analysis that adjusted for available case-mix and payer-mix measures. There was also no strong evidence that Maryland hospitals experienced lower margin volatility during COVID-19 than Massachusetts hospitals. Overall, these results suggest that global budgets may alter the mechanisms by which hospitals generate revenue per case without producing substantial, detectable changes in profitability or financial stability during the pandemic period.

## 1. Introduction

### 1.1. Problem Framing

The financial sustainability of hospitals remains a policy issue because they are essential to ensuring access to care, emergency care, and market capacity. Under the traditional fee-for-service (FFS) reimbursement model, hospital revenues depend critically on utilization, exposing hospitals to significant financial risk when inpatient admissions or outpatient volumes decline. The coronavirus disease 2019 pandemic offered a clear test for hospital financing dependent on utilization, with substantial risks of financial distress documented.^1,2^

Prospective revenue models have therefore proven appealing for their potential to break the link between volume and revenue, increasing revenue predictability. Yet adopting prospective revenue streams does not necessarily translate into higher profits or lower per-patient revenues. Hospital operations may shift under fixed-revenue constraints through changes in service mix, coding intensity, discharge rates, or other means, affecting average revenue per discharge regardless of the impact on margins. Prior literature on prospective, capitated, and budget-based payment models has also raised concern that hospitals may respond through documentation intensity, service-line reallocation, or selective concentration in higher-revenue cases, even when overall revenue growth is constrained. Such mechanisms can alter revenue per discharge without necessarily improving profitability. The present study cannot directly distinguish among these mechanisms, but the revenue-per-discharge outcome should be interpreted in that broader conceptual context. As such, the analysis of financial reforms should include several key performance dimensions: profitability (margins), revenue intensity (revenue per discharge), and resilience to shocks.

### 1.2. Policy Context

Maryland’s All-Payer Model serves as the leading example of nationwide hospital rate regulation combined with global budgets in the United States. Since 2014, under a waiver from the Centers for Medicare & Medicaid Services (CMS), Maryland has implemented an all-payer global budget regulatory system for hospitals within its jurisdiction.^3,4^ Maryland adopted this global budget model to shift away from volume-based hospital payments, enhance cost control, and provide more predictable hospital revenue under its all-payer rate-setting framework. In this new approach, hospitals no longer depend on FFS payment models; instead, they set annual revenue caps prospectively.

Early analysis of Maryland’s All-Payer Model has largely emphasized cost and utilization outcomes as anticipated in cost-containment reform. For example, Beil et al have identified savings in Medicare payments and certain utilization metrics in the first 3 years of the reform’s implementation, while Roberts et al considered the first 2 years of the hospital global budgets and failed to detect program-driven utilization change.^5,6^ Morrison et al later reported relatively slower increases in Medicare and commercial hospital payments in Maryland compared with control areas, with outpatient payments accounting for this trend.^7^ In sum, early analyses of the Maryland all-payer model highlighted the initiative’s experimental nature and its relevance to policy discussions but focused primarily on cost and utilization outcomes, leaving hospitals’ financial outcomes largely unexplored.

Another set of studies has analyzed the operations of hospitals operating under the reform. For example, Kilaru et al noted that Maryland’s all-payer payment structure helped to create conditions for effective planning and more predictable financing, at the same time posing additional administrative and operational challenges.^8^ In turn, Perry et al identified the key strategies behind the successful performance of Maryland hospitals under their global budgets, namely, physician employment, clinically oriented patient education, use of health information exchanges, physician alignment, and custom analytics.^9^ More recent policy analyses have also highlighted the importance of adaptations to specialty care within hospital global budgets.^10^ In addition to the existing all-payer model, Maryland has developed another innovative program, called Total Cost of Care, that aims to strengthen hospital accountability.^11-13^

### 1.3. Research Gap

Despite Maryland’s All-Payer Model being among the best-understood hospital payment reforms in the United States, knowledge gaps still exist.

First, there is a lack of comparative evidence on hospital finances under the all-payer model past 2014. Existing literature focuses mostly on spending and utilization as well as hospital implementation of the reform, but does not address hospital finances past the early post-reform period.^5-7,11^ Specifically, studies on hospital adaptation under the reform show that Maryland hospitals have modified their behavior, but it is unclear whether these changes have resulted in margin increases, higher revenues per discharge, and greater financial stability.

Second, dynamic validation techniques continue to play a crucial role in comparative analyses. Conventional static difference-in-differences (DiD) models cannot accurately reflect evolving treatment effects or divergent trends prior to treatment assignment. To ensure robust results, event-study diagnostics and placebo falsification tests should be conducted.^14-16^

Third, shock period stability analysis has yet to be conducted in Maryland versus a comparison group. While Maryland’s global budgets have often been viewed as a potential mechanism to reduce reliance on utilization-based revenue and increase revenue predictability, relatively little comparative research has examined whether the reform stabilized hospital margins during the COVID-19 shock period of 2020-2022. Recent research has confirmed high financial risk and variability during this period for different types of hospitals, but has paid less attention to whether global budgets have helped to improve financial stability.^1,2^

Using a matched hospital-year panel for Maryland and Massachusetts based on RAND’s HCRIS-derived cost report data for 2010-2023,^17^ this study evaluates 3 hospital financial outcomes: total margin, log revenue per discharge, and margin volatility during the COVID-19 period (2020-2022). The study asks whether Maryland’s 2014 implementation of all-payer global budget revenue (GBR) was associated with differential changes in hospital margins (RQ1) and revenue per discharge (RQ2) relative to Massachusetts, and whether Maryland experienced different margin volatility during the COVID-19 period (RQ3). Based on the policy design and prior literature, the analysis expects a positive post-2014 differential effect on revenue per discharge, but no substantial differential effect on total margins or on COVID-period margin volatility. To examine these questions, the study applies difference-in-differences methods with hospital and year fixed effects, hospital-clustered standard errors, event-study diagnostics, and a placebo falsification test.^14-16^

## 2. Background

### 2.1. Maryland GBR Mechanics (Why Revenue per Discharge Might Move)

Maryland’s All-Payer Model implemented hospital global budgets beginning in 2014, replacing a volume-driven revenue model with a prospective revenue target regulated through the HSCRC under a CMS waiver.^3,4^ In practice, GBR changes the hospital’s problem from “maximize billable volume” to “manage within a revenue constraint while meeting quality and accountability expectations.” This shift has two direct implications for the outcomes in this paper: it can change unit-revenue measures (e.g., revenue per discharge) even if it does not raise margins, and it may reduce exposure to short-run volume shocks without guaranteeing lower volatility in accounting margins.

Why might revenue per discharge increase under a revenue cap? Several mechanisms are plausible and are consistent with the logic of prospective payment:

• **Intensity/service-mix responses:** When overall revenue growth is constrained, hospitals have incentives to protect revenue by shifting toward a higher-acuity case mix in inpatient admissions, increasing service intensity per stay, or re-optimizing coding/documentation. These changes can increase average revenue per discharge even if aggregate revenue is regulated.
• **Rebalancing across settings and lines of business:** GBR incentives can encourage hospitals to reduce avoidable utilization and manage volume; however, hospitals may respond by altering service composition (e.g., focusing on profitable lines of business within regulatory constraints). This can change unit-revenue measures even when total revenue is held constant.
• **Margins need not rise:** Even if unit revenue increases, margins may remain stable if costs rise in parallel (labor, supplies, capital) or if efficiency gains offset revenue changes. Prior evaluations of Maryland’s global budgets emphasize cost/utilization outcomes and do not imply systematic margin expansion as an intended goal of GBR.^5^ Qualitative accounts similarly stress operational adaptation and administrative demands rather than windfall profitability.^8^

### 2.2. Why Massachusetts Is a Defensible Comparator

A credible comparator should (i) share broad structural features that affect hospital finance trends and (ii) lack the treated policy mechanism. Massachusetts offers a valuable comparison for Maryland because both states are high-income U.S. jurisdictions with advanced hospital systems, high insurance coverage rates, and mature sectors featuring large integrated delivery systems and academic medical centers.^18^ Both are embedded in a similar macro-regional and national financing environment, including exposure to the same federal policies, economic cycles, and shocks (e.g., macroeconomic trends, ACA expansions, and COVID-era disruptions). This comparability strengthens identification, as the main estimand in this study is the differential change in outcomes for Maryland hospitals after 2014 relative to a non-GBR state facing parallel external pressures.

Crucially, Massachusetts does not operate a statewide, all-payer global budget revenue (GBR) system analogous to Maryland’s HSCRC-regulated model. Instead, Massachusetts largely retains fee-for-service (FFS) payments and emphasizes transparency, cost-growth benchmarks, and voluntary cost-control initiatives through payer/provider contracting rather than mandatory rate-setting. Massachusetts also serves as a useful policy counterpoint: it is often described as “reform-oriented,” with experience with global payment approaches (e.g., ACO contracts) that can affect spending and quality under certain designs, but these differ fundamentally from Maryland’s statewide all-payer regulation. As a result, Massachusetts serves as a pragmatic benchmark, a high-capacity, reform-active environment without Maryland’s distinctive all-payer GBR infrastructure.^19^ The contrast between Maryland’s rate-regulated, all-payer system and Massachusetts’s benchmark-driven, market-based approach provides a natural setting to examine whether global budgeting is associated with distinct trajectories in hospital margins and prices.

### 2.3. Conceptual Pathways

**Figure 1.**
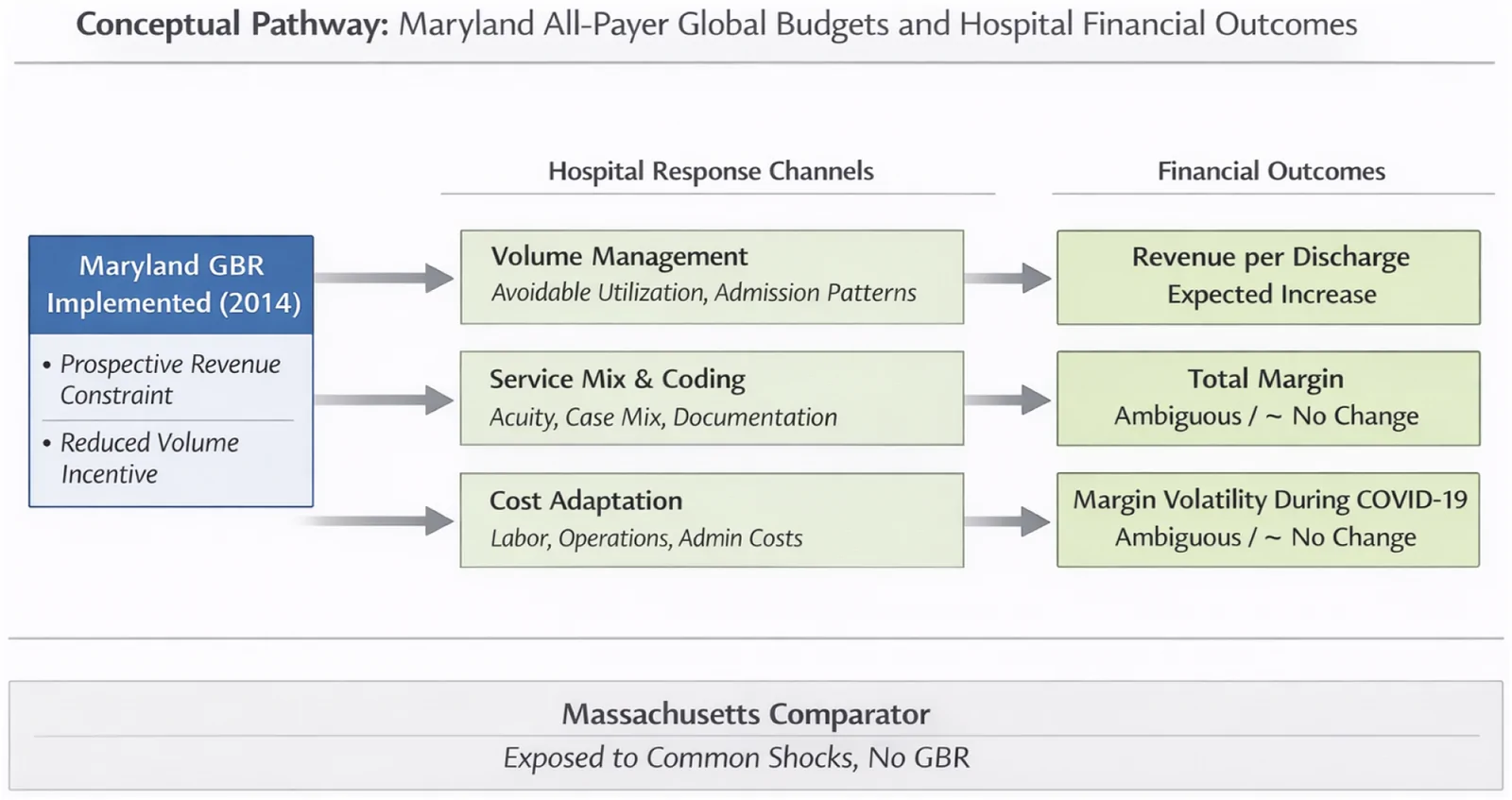
Conceptual pathway linking Maryland’s all-payer global budgets to hospital financial outcomes (Maryland vs Massachusetts) *Note.* GBR indicates global budget revenue; HSCRC, Health Services Cost Review Commission; CMS, Centers for Medicare & Medicaid Services

Figure 1 summarizes the conceptual channels through which GBR could affect unit revenue (revenue per discharge), margins, and margin volatility relative to a non-GBR comparator.

Maryland’s 2014 global budget revenue model introduced prospective hospital revenue targets under the oversight of the Health Services Cost Review Commission and a Centers for Medicare & Medicaid Services waiver.^3,4^ The model may influence hospital behavior through reduced reliance on marginal admissions, stronger incentives to manage avoidable utilization, shifts in service mix and coding intensity, redesign of care pathways, and operational cost adaptation.^5,8^

These responses could increase revenue per discharge if changes in case mix, service intensity, or documentation predominate. Total margins, however, may remain unchanged if costs rise alongside revenues or if the regulatory framework limits sustained increases in profitability. During the COVID-19 period, prospective revenue arrangements may have buffered hospitals against volume-related revenue losses, although margin volatility could still reflect cost increases, federal relief payments, and differences in pandemic caseloads.^1,2^ Massachusetts therefore provides a fee-for-service comparison exposed to similar secular and pandemic-related pressures but without Maryland’s statewide all-payer global budget structure.

These conceptual pathways motivate the study’s focus on log revenue per discharge, total margin, and margin volatility as distinct but related dimensions of hospital financial performance under global budgets.^3-6^

## 3. Methods: Data and Study Design

### 3.1. Data Construction, Analytic Sample, and Reporting Guideline

Hospital financial outcomes were obtained from RAND Hospital Cost Report Information System (HCRIS)-derived files, which standardize Medicare hospital cost-report data into longitudinal analytic variables suitable for hospital finance research.^17^ Time-varying provider characteristics were obtained from the Centers for Medicare & Medicaid Services (CMS) Provider of Services (POS) File—Hospital & Non-Hospital Facilities, which contains certification and facility characteristics linked to the CMS Certification Number (CCN).^20^

The analytic panel was constructed by merging the RAND hospital-year file with the POS file at the CCN-by-fiscal-year level using a 1:1 matched-only merge, retaining only matched observations. This process yielded 2,261 matched hospital-year observations from fiscal years 2010 to 2023. The resulting panel was unbalanced, reflecting variation in hospital reporting across years. A hospital panel identifier (*hid*) was constructed from the CCN where needed.

These mechanisms motivate the directional expectations used in this manuscript: H1 (positive effect on revenue per discharge) is plausible under behavioral and accounting responses to prospective revenue constraints, while H2 (null effect on margins) is plausible if cost dynamics and regulatory design prevent systematic profit expansion.^3-5,8^

Maryland’s 2014 statewide implementation of all-payer global hospital budgets defined the treatment contrast. The main treatment term was specified as an interaction between an indicator for Maryland and an indicator for the post-2014 period. This manuscript was prepared in accordance with the Strengthening the Reporting of Observational Studies in Epidemiology (STROBE) guideline, and the completed checklist is provided in the Supplementary file.^21^

Patterns of outcome missingness by state and fiscal year are reported in Appendix Table S1.

The analytic sample was restricted to hospital-year observations from Maryland and Massachusetts between 2010 and 2023 that could be matched 1:1 using CCN and fiscal year across the RAND HCRIS-derived file and the CMS POS file. Observations without valid matches, outside the study states or study period, or lacking the information required to construct outcome-specific measures were excluded from the relevant analyses.

### 3.2. Statistical Analysis

This study used a comparative interrupted-panel design implemented as a two-way fixed-effects DiD framework to estimate the association between Maryland’s 2014 implementation of all-payer global hospital budgets and hospital financial outcomes relative to Massachusetts. Hospital fixed effects and year fixed effects were included to account for time-invariant hospital characteristics and common secular shocks, respectively. Standard errors were clustered at the hospital level to account for within-hospital serial correlation and heteroskedasticity, consistent with recommended practice for DiD analyses.^15^

To address **RQ1**, concerning whether Maryland’s global budget revenue (GBR) model changed total hospital margins relative to Massachusetts after 2014, we estimated a fixed-effects DiD model with total margin as the dependent variable and the interaction between an indicator for Maryland and an indicator for the post-2014 period as the principal estimand. To address **RQ2**, concerning whether Maryland’s GBR model changed log inpatient revenue per discharge relative to Massachusetts after 2014, we estimated an analogous fixed-effects DiD model using log revenue per discharge as the dependent variable and the same Maryland × post-2014 interaction term as the coefficient of interest.

To address **RQ3**, concerning whether Maryland experienced different margin volatility during the COVID-19 period, we estimated a fixed-effects panel model in which the outcome was margin volatility, defined as the absolute year-over-year change in total margin for consecutive-year observations, and the principal term of interest was the interaction between Maryland and the COVID-period indicator (2020–2022).

To assess identification, we estimated an event-study specification to examine pre-policy trends and post-policy dynamics and conducted a placebo test using a false intervention date in 2012 restricted to the pre-policy period.^14-16^ Because financial outcomes derived from cost reports may be sensitive to extreme values, sensitivity analyses using winsorized volatility measures were also performed. All analyses were conducted in Stata/SE 18.

No formal a priori power calculation was conducted because the study used the full available matched hospital-year panel from the selected data sources and study period.

### 3.3. Outcomes and Variable Definitions

Three outcomes were prespecified to align with the study questions on level effects (margins; revenue per discharge) and stability during the COVID period.

#### 3.3.1. Total Margin (Profitability)

Total margin was measured using RAND’s standardized cost-report variable *Totalmargin* (total margin; net income divided by total revenue). This outcome is a standard measure in hospital finance research, used to summarize profitability and financial viability. Because total margin can exhibit heavy tails due to accounting anomalies, extraordinary items, and reporting outliers in cost reports, sensitivity analyses were designed to assess the robustness of the conclusions to winsorization strategies (described below).^17^

#### 3.3.2. Log Inpatient Revenue per Discharge (Price Proxy/Unit Revenue)

The primary “price proxy” was the natural log of inpatient revenue per discharge (ln_price_per_discharge), operationalized as inpatient revenue divided by inpatient discharges, then log-transformed. This measure is interpreted as a unit-revenue metric, capturing changes in revenue intensity per inpatient stay. It does not isolate transaction prices at the service-line level but is widely used as a tractable proxy in cost report–based analyses when payer-specific prices are unavailable.^17^

Payer mix and case mix were considered as potential confounders, particularly for the revenue-per-discharge outcome. However, consistently harmonized measures with adequate completeness across the full matched 2010-2023 hospital-year panel were not available for inclusion in the primary specification. The preferred models, therefore, relied on hospital fixed effects and year fixed effects, with residual time-varying confounding acknowledged as a limitation.

#### 3.3.3. Margin Volatility (Stability), Including the COVID Stress-Test

Volatility was defined as the absolute 1-year change in total margin for consecutive-year observations:

$$|\Delta {\text{margin}}_{it}|=|{\text{margin}}_{it}-{\text{margin}}_{i,t-1}|$$

constructed only when hospital *i* reported total margin in both year *t* and *t−1*, and years were consecutive. This design intentionally measures short-run instability rather than long-run trend shifts. To assess whether global budgets functioned as a “shock absorber” during the pandemic—an empirically important stress test for volume-sensitive payment systems—an indicator for the COVID period (2020–2022) was used to estimate a Maryland differential in volatility during COVID relative to Massachusetts.^1,2^

Outcome availability differed by measure due to missing cost report items and the consecutive-year requirement for volatility. Therefore, descriptive tables report **state-specific non-missing counts** for each outcome (rather than reusing the full merged N), and all regressions were estimated on the maximal non-missing sample for the dependent variable under study.

In an additional restricted sensitivity analysis for 2010–2019, available case-mix and payer-mix measures were included to assess the robustness of the revenue-per-discharge findings.

### 3.4. Identification Strategy: Comparative Interrupted Panel (DiD With Fixed Effects)

The primary causal estimand was the average differential change in Maryland hospital outcomes after implementation of statewide all-payer global budgets in 2014, relative to contemporaneous changes in Massachusetts.

#### 3.4.1. Policy Break (Treatment Timing)

Maryland’s all-payer global budget model began in 2014, motivating the pre/post definition used in the main DiD specification.^3,4^ The treatment indicator was defined at the state level (Maryland vs Massachusetts), and the post-policy indicator was defined as fiscal year ≥ 2014. The interaction term (**MD × Post2014**) captured the DiD effect.

#### 3.4.2. Main DiD Model

For hospital *i* in year *t*, the core specification was:

$$Y_{it}=\alpha_{i}+\lambda_{t}+\beta \left({\text{MD}}_{i}\times {\text{Post}2014}_{t}\right)+\varepsilon_{it}$$

where $\alpha_{i}$ are hospital fixed effects and $\lambda_{t}$ are year fixed effects. The fixed effects absorb all time-invariant differences between hospitals (e.g., baseline teaching status, persistent market position) and all common shocks by year (e.g., national policy, macroeconomic conditions, pandemic-era national changes).^15,16^ The coefficient β is interpreted as the average post-2014 differential change in Maryland relative to Massachusetts, net of hospital-invariant and year-common confounding.

This framework aligns with best-practice guidance emphasizing (i) a clearly defined intervention time, (ii) appropriate fixed effects, and (iii) cluster-robust inference at the unit of treatment assignment (hospital).^15,16^

#### 3.4.3. Interrupted-Panel Framing

Although DiD terminology is used throughout, the design also reflects an interrupted-panel logic: Maryland experienced a clear statewide policy break, and Massachusetts provides the counterfactual trajectory absent all-payer global budgets. This framing is particularly appropriate when the intervention occurs at the state level and hospital outcomes may evolve gradually rather than discontinuously.

### 3.5. Diagnostics and Validity Checks

Because DiD validity depends on the plausibility of parallel trends and on avoiding overinterpretation of a single average post-indicator, diagnostics were specified as core components of the design rather than add-ons.^14-16^

#### 3.5.1. Event Study (Pre-Trends and Dynamics)

An event-study specification was estimated for the total margin to test for evidence of differential pre-trends and to characterize post-implementation dynamics. Following standard event-study practice, relative time indicators around 2014 were interacted with the Maryland indicator, with the pre-policy year omitted as the baseline. Event studies also address a key limitation of static DiD: a single post-coefficient can mask dynamic adjustment and the heterogeneous timing of effects.^14^ The primary diagnostic interpretation focuses on whether pre-policy relative-time coefficients are close to zero (supporting parallel trends in the pre-period) and whether post-policy coefficients show a consistent pattern.

#### 3.5.2. Placebo Test (Fake Treatment Timing)

A placebo DiD was estimated using a false policy date (2012) restricted to the pre-period (through 2013). A null placebo estimate increases confidence that the observed post-2014 differences are not artifacts of spurious timing or of unrelated shocks that affected the two states differently prior to the true intervention window.^15,16^

#### 3.5.3. COVID-Period Volatility Model (Stress Test)

To evaluate the hypothesis that global budgets may stabilize finances during volume shocks, volatility models estimated a Maryland differential during COVID (2020–2022) using an interaction (**MD × COVID**) in a fixed-effects panel. This approach directly addresses the empirical concern that fee-for-service environments may exhibit larger revenue and margin swings during major utilization shocks.^1,2^

#### 3.5.4. Sensitivity Analyses for Outliers (Winsorization)

Given the heavy-tailed nature of financial ratio outcomes, particularly volatility measures derived from differences, winsorized variants of the volatility outcome were estimated (e.g., trimming at extreme percentiles) to ensure that inference was not driven by a small number of extreme observations. This is a pragmatic robustness step in cost-report–based financial analyses, where outliers can reflect reporting anomalies rather than true economic variation.^17^

As a sensitivity analysis, the main difference-in-differences models were re-estimated in a restricted sample of hospitals observed in both the pre-2014 and post-2014 periods to assess whether changing sample composition across periods materially influenced the findings.

### 3.6. New Dataset Documentation Note

The POS file used for the merge is updated quarterly and explicitly describes certification, ownership, location, and related provider characteristics, organized by CMS Certification Number. A new POS file covering additional facility types was introduced beginning Q4 2023, and the dataset metadata was updated in January 2026.^20^

### 3.7. Software

Statistical analyses were conducted in **Stata/SE 18** (StataCorp LLC, College Station, TX) using two-way fixed-effects panel regressions with hospital-clustered standard errors. Output tables and figures were generated using Stata add-on packages (**estout/esttab**, **coefplot**).

## 4. Results

### 4.1. Descriptive Statistics and Trends

Table 1 describes the merged analytic panel of hospitals in Maryland (MD) and Massachusetts (MA) from 2010–2023 (2,261 matched hospital-year observations). Massachusetts contributes 1,431 hospital-years (63.3%) and Maryland 830 (36.7%). Outcome availability varies by measure: total margin is observed in 1,995 hospital-years (MA: 1,263; MD: 732), ln(revenue per discharge) in 2,044 hospital-years (MA: 1,301; MD: 743), and the volatility outcome, absolute one-year change in total margin for consecutive years, in 1,833 hospital-years (MA: 1,161; MD: 672).

**Table 1.** Analytic Sample and Outcome Availability (MD vs MA), 2010–2023

Item	Massachusetts (MA)	Maryland (MD)	Total
Hospital-years in merged analytic file	1,431	830	2,261
Years covered	2010–2023	2010–2023	2010–2023
Outcome availability: total_margin (non-missing hospital-years)	1,263	732	1,995
Outcome availability: ln_price_per_discharge (non-missing hospital-years)	1,301	743	2,044
Outcome availability: abs_d_margin (non-missing hospital-years)	1,161	672	1,833

*Notes.* Counts are hospital-year observations in the merged analytic file. abs_d_margin is defined only for **consecutive**hospital-year pairs (requiring total_margin in years *t* and *t−1,* with no gaps), so its N is smaller by design.

Appendix Table S1 reports outcome missingness by state and fiscal year and indicates that missingness affected both states throughout the study period, with no evidence of a large or persistent divergence in magnitude across states.

**Figure 2.**
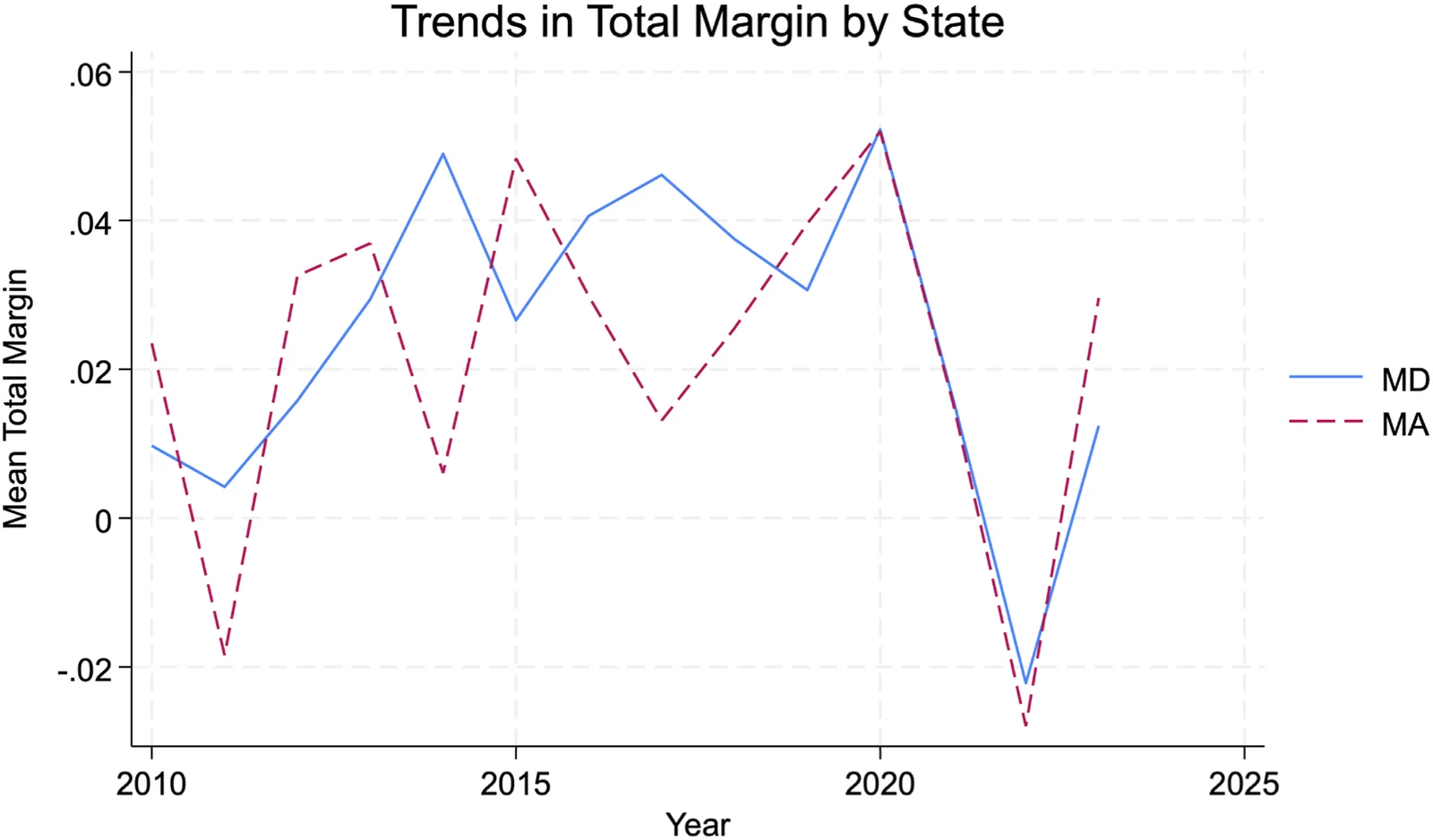
Trends in total margin by state, 2010–2023 (Maryland vs Massachusetts) *Note.* Lines show state-year means of hospital total margin from the merged analytic file (RAND HCRIS-derived measures). Maryland’s all-payer global budget revenue model began in 2014

Figure 2 presents state-year trends in mean total margin for Maryland and Massachusetts from 2010–2023. Visual inspection suggests broadly similar long-run trajectories, with year-to-year variation in both states and no sharp or sustained post-2014 divergence consistent with a large, immediate profitability shift under global budgets.

**Figure 3.**
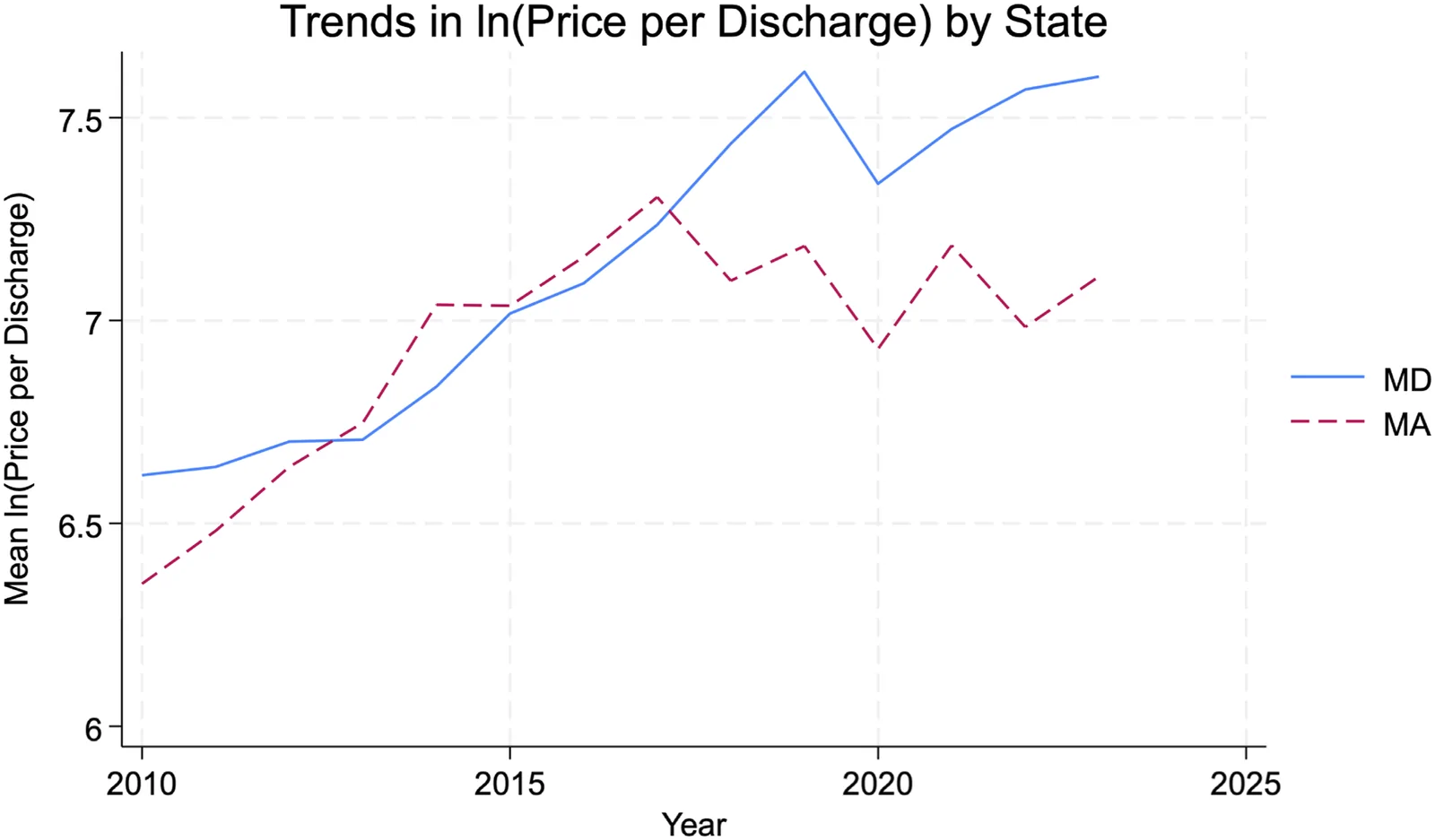
Trends in ln(revenue per discharge) by state, 2010–2023 (Maryland vs Massachusetts) *Note.* Lines show state-year means of hospital-year ln(revenue per discharge) from the merged analytic file (RAND HCRIS-derived measures). Maryland’s all-payer global budget revenue model began in 2014

Figure 3 presents state-year trends in mean ln(revenue per discharge). The series diverge after the mid-2010s, with Maryland exhibiting higher ln(revenue per discharge) levels than Massachusetts in later years, consistent with the regression evidence of a positive post-2014 differential change.

These plots summarize raw (unadjusted) means and do not account for hospital fixed effects or national shocks captured in the DiD framework.

### 4.2. Main DiD Estimates

Table 2 reports the fixed-effects DiD estimates with hospital and year fixed effects and standard errors clustered at the hospital level. [15,16] The coefficient of interest in each model is the interaction **MD × Post2014 (md_post2014)**.

**Table 2.** Main DiD Estimates: Effect of Maryland Post-2014 GBR on Financial Outcomes (Hospital FE, Year FE)

Outcome	Treatment term	β	SE (clustered by hospital)	p-value	N (hospital-years)	Hospitals (clusters)
Total margin	md_post2014	0.0213	0.0177	0.232	1,995	160
ln(Revenue per discharge)	md_post2014	0.2872	0.0946	0.003	2,044	168

*Notes.* **Interpretation (log outcome):** For ln_price_per_discharge, the percent change is **exp(β) − 1**. Here, exp(0.287)−1 ≈ **+33.3%** (95% CI ≈ **+10.6% to +60.6%**).

#### 4.2.1. RQ1 (Margins)

In the total margin model, the estimated post-2014 differential for Maryland relative to Massachusetts was positive but not statistically distinguishable from zero (**β = 0.021**, SE = 0.018; **p = 0.232**) (Table 2). This result does not support a detectable post-2014 change in operating profitability attributable to global budgets in the Maryland–Massachusetts comparison. The evidence is therefore most consistent with no measurable average margin effect.

#### 4.2.2. RQ2 (Revenue per Discharge)

In the ln(revenue per discharge) model, the post-2014 differential for Maryland relative to Massachusetts was positive and statistically significant (**β = 0.287**, SE = 0.095; **p = 0.003**) (Table 2). Interpreted in log points, this corresponds to an approximate **33% increase** in revenue per discharge for Maryland relative to Massachusetts after 2014 exp(0.287)−1. In the primary specification, this pattern is consistent with higher unit revenue intensity under global budgets.

Sensitivity analyses restricted to hospitals observed in both the pre- and post-2014 periods yielded substantively identical findings. The post-2014 differential remained null for total margin (β = 0.021, p = 0.233) and positive and statistically significant for log revenue per discharge (β = 0.287, p = 0.003).

In an additional restricted sensitivity analysis for 2010–2019 that adjusted for available case-mix and payer-mix covariates, including Medicare case-mix index, Medicare inpatient day share, and Medicaid inpatient day share, the post-2014 Maryland differential for log revenue per discharge was attenuated and no longer statistically significant (β = 0.030, p = 0.513). This pattern suggests that the main revenue-per-discharge finding is sensitive to adjustment for observed patient composition and payer structure in the restricted sample.

### 4.3. Event Study (Identification, Diagnostics, and Dynamics for Margins)

**Figure 4.**
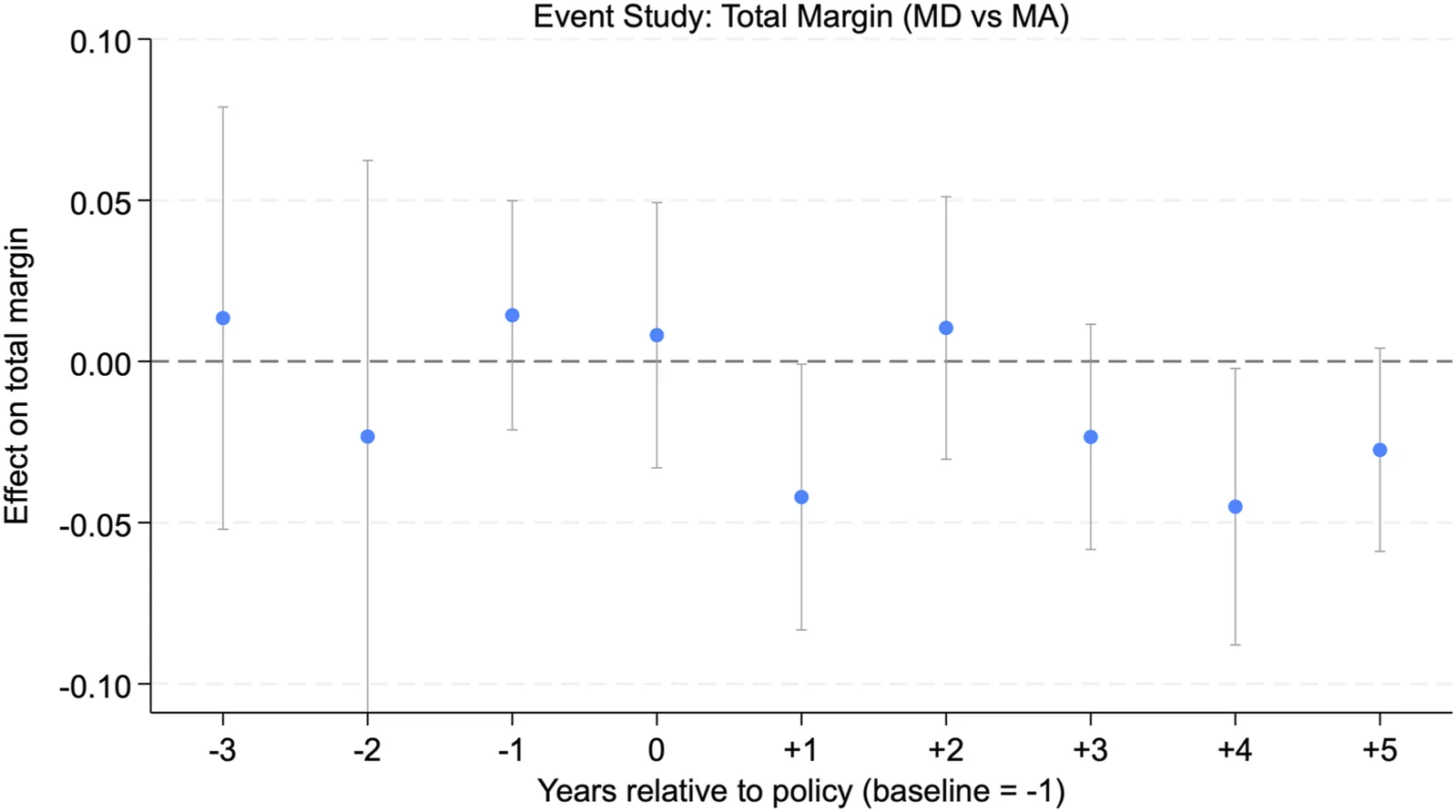
Event-study estimates for total margin: Maryland–Massachusetts differential around 2014 global budgets *Note.* Points plot the estimated Maryland-minus-Massachusetts differential in total margin at each event time (relative to 2014), with 95% confidence intervals. Estimates are from a hospital fixed-effects model with fiscal-year fixed effects and hospital-clustered standard errors; the omitted reference period is relative year −1 (the year immediately prior to implementation)

Figure 4 displays the event-study estimates for total margin, expressed as the **Maryland–Massachusetts differential** at each event time relative to the omitted baseline year (baseline = −1). Pre-policy coefficients (e.g., −3 and −2) are close to zero and statistically non-significant, providing supportive (though not definitive) evidence that differential pre-trends are not apparent for margins in the event-time window examined (Figure 4).

Post-policy estimates show some negative coefficients at select horizons, including statistically notable negatives at +1 and +4 in the event-study output. However, confidence intervals are wide at multiple horizons and the primary DiD estimate for margins is imprecise. Therefore, dynamic estimates are interpreted as **diagnostic and descriptive of potential heterogeneity over time**, rather than as definitive effect sizing.^14^ The main conclusion remains anchored to the static DiD estimate in Table 2: no statistically detectable average post-2014 margin effect.

### 4.4. COVID-Period Margin Volatility (RQ3/H3)

Table 3 reports fixed-effects models for margin volatility, measured as the absolute year-to-year change in total margin between consecutive years. The key coefficient is the interaction **MD × COVID (md_covid)** for 2020–2022.

**Table 3.** COVID-Period Volatility (RQ3): Differential Change in Margin Volatility in Maryland vs Massachusetts, 2020–2022

Volatility outcome	Specification	Treatment term	β	SE (clustered)	p-value	N	Hospitals
abs_d_margin	Raw	md_covid	−0.0180	0.0128	0.161	1,833	160
abs_d_margin	Winsorized 1/99	md_covid	−0.0075	0.0094	0.430	1,833	160
abs_d_margin	Winsorized 5/95	md_covid	0.0009	0.0058	0.871	1,833	160

*Notes.* Covid = 1[fiscal_year ∈ (2020,2021,2022)], md_covid = 1[MD]×covid. abs_d_margin = abs(total_margin − L.total_margin) for consecutive years only. Models include hospital FE and fiscal-year indicators; SE clustered by hospital.

The estimated Maryland differential in volatility during COVID was negative but not statistically significant (**β = −0.018**, **p = 0.161**) (Table 3), indicating **no evidence of differential volatility reduction** in Maryland relative to Massachusetts during the COVID period. Winsorized variants of the volatility outcome (constructed to reduce sensitivity to extreme changes) yielded similarly null interaction estimates, reinforcing the conclusion that results are not driven by outliers.

### 4.5. Placebo Test (Validity Check)

Table 4 reports the placebo DiD estimate using a fake treatment date in 2012, restricted to the pre-policy period. The placebo interaction was near zero and not statistically significant (**β = −0.008**, **p = 0.801**) (Table 4). This null finding supports the interpretation that the main results are unlikely to be driven by a spurious pre-period break unrelated to the 2014 implementation.

**Table 4.** Identification/Validity Checks: Event Study (Pre-Trends) and Placebo (Fake Treatment). (A). Event Study (Total Margin): MD–MA Differential by Event Time (Baseline = −1), (B). Placebo DiD (Fake Treatment Year 2012; Estimated on Pre-Period Through 2013)

Relative year to 2014 (baseline = −1)	Coefficient	SE	p-value	Interpretation
−3	0.0134	0.0332	0.686	Pre-trend: null
−2	−0.0233	0.0434	0.592	Pre-trend: null
−1	0 (omitted)	—	—	Baseline year
0	0.0081	0.0208	0.697	Immediate shift: null
+1	−0.0421	0.0209	0.045	Post: negative (borderline)
+2	0.0104	0.0206	0.616	Post: null
+3	−0.0234	0.0177	0.187	Post: null
+4	−0.0451	0.0217	0.039	Post: negative
+5	−0.0275	0.0160	0.087	Post: negative (borderline)

Outcome	β (MD × PlaceboPost2012)	SE	p-value	Interpretation
Total margin	−0.0081	0.0319	0.801	No “effect” when policy timing is fake → supports design validity

**Outcome:** total_margin. **Model:** hospital FE + fiscal-year indicators; SE clustered by hospital (hid). **Displayed coefficients:** Maryland–Massachusetts differential at each event time *relative to the omitted baseline year (−1)*.

RQ1/H1 (Margins/Stability):

The pre-period leads (−3 and −2) are both null, supporting the plausibility of parallel trends for total margin without establishing it. Negative coefficients at +1, +4, and +5 suggest possible short-to medium-run dips, though these are not consistently aligned with the null main DiD estimate for margins.

Notes: Coefficients are from the event-study specification using relative-year-by-Maryland interaction terms and are expressed in margin units (approximately percentage points).

**Outcome:** total_margin. **Sample restriction:** fiscal years ≤ 2013. **Model:** hospital FE + fiscal-year indicators; SE clustered by hospital (hid).

*Notes.*Event-study estimates are from a fixed-effects panel model with hospital and fiscal-year indicators and hospital-clustered standard errors. Coefficients represent Maryland–Massachusetts differentials at each event time relative to the omitted baseline (−1). The placebo test assigns a false treatment start in 2012 and estimates the model using only pre-policy years through 2013.

## 5. Discussion

### 5.1. Principal Findings

This comparative interrupted panel analysis examined whether Maryland’s all-payer global budget revenue (GBR) model—implemented in 2014—was associated with differential changes in hospital financial outcomes relative to Massachusetts from 2010–2023. Three findings stand out.

First, the primary model suggested that Maryland’s GBR implementation was associated with a statistically significant increase in ln(revenue per discharge) relative to Massachusetts. However, in an additional restricted sensitivity analysis for 2010–2019 that adjusted for available case-mix and payer-mix covariates, including Medicare case-mix index, Medicare inpatient day share, and Medicaid inpatient day share, the post-2014 Maryland differential for log revenue per discharge was attenuated and no longer statistically significant (β = 0.030, p = 0.513). Interpreted cautiously, the primary model suggests a higher average inpatient revenue per discharge in Maryland after 2014, although this association was sensitive to additional adjustment for available case-mix and payer-mix measures. This pattern is directionally compatible with theory and qualitative accounts suggesting that prospective revenue environments can change hospitals’ operational responses, including service mix, coding, and intensity decisions, even when overall revenue growth is regulated.^3-5,8,11^

Second, the results do not reject H2: there is no statistically precise evidence that GBR produced a discrete shift in total margin relative to the comparator. The appropriate interpretation is not “no effect” but rather “no detectable average margin change,” given the observed variance, heavy tails in margins, and the design’s identifying assumptions. The result remains consistent with early evaluations that emphasize utilization and spending effects rather than clear changes in profitability, and with GBR’s policy intent to constrain revenue growth and discourage volume-driven surplus generation.^3-5,11^

Third, the findings do not reject H3: during the COVID period (2020–2022), Maryland does not exhibit a statistically distinct change in year-to-year margin volatility relative to Massachusetts. This is an important negative result given the policy narrative that prospective payment arrangements may buffer hospitals against volume shocks. COVID-era evidence documents substantial financial stress and heterogeneity in hospital resilience, but comparative evidence separating payment structure from broader market and policy factors remains limited.^1,2^ The present results indicate that any differential volatility buffering attributable to GBR is not clearly detectable in this two-state fixed-effects comparison.

One plausible explanation is that pandemic-era federal relief, including CARES Act Provider Relief Fund payments and related support, may have dampened volatility in fee-for-service settings such as Massachusetts, thereby reducing the observable contrast that Maryland’s prospective global budgets might otherwise have produced.

### 5.2. Interpretation That Remains Within the Data

A central interpretive contribution is the combination of higher inpatient revenue per discharge (a revenue-intensity proxy) with no detectable corresponding margin shift. This is not contradictory. Revenue per discharge can rise while margins remain stable if costs, constraints, and accounting totals adjust in parallel. Under GBR, hospitals operate within a regulated revenue framework but still face internal incentives related to case mix, throughput management, staffing, and service line composition; some of these mechanisms may increase unit revenue measures while leaving net margins unchanged.^3-5,8,11^

The additional restricted-sensitivity analysis indicates that this revenue-intensity finding is not entirely independent of observable variation in case mix and payer structure and therefore should not be interpreted as a pure payment-model effect.

At the same time, the revenue-per-discharge outcome must be treated as a proxy, not a direct measure of negotiated or regulated prices. Hospital cost report revenue measures reflect a combination of accounting practices, payer mix, and service intensity; therefore, the analysis supports statements about revenue intensity rather than definitive claims about “prices.” This distinction is important for external validity and policy translation, particularly when comparing a rate-regulated state to a benchmark-oriented fee-for-service environment.^4,11^

The event-study and placebo checks strengthen interpretability but do not eliminate uncertainty. The placebo analysis does not indicate spurious pre-period breaks, consistent with design validity. The event-study estimates provide supportive diagnostics for the plausibility of pre-trends, though uncertainty in several periods warrants modest claims rather than dynamic overinterpretation.^14-16^

### 5.3. Policy Implications

These findings have implications for states and federal initiatives evaluating prospective payment or budget-based approaches.

**1. Monitor intensity and service-mix responses.** If global budgets are associated with higher revenue per discharge without margin inflation, attention should shift to whether unit-revenue changes reflect desirable efficiency, coding shifts, or service substitution. Program governance may require complementary monitoring of case mix, coding, and service line patterns to prevent unintended incentives that increase revenue intensity without improving value.^3-5,8,11^
**2. Avoid assuming “automatic financial stabilization.”** Prospective models are often described as stabilizing during shocks, yet the COVID volatility test does not show statistically clear differential buffering in Maryland relative to Massachusetts. The implication is not that stabilization does not occur, but that it should be treated as an **empirical question** that may depend on implementation details, external relief policies, and hospital baseline risk exposure.^1,2,4,11^
**3. Support careful scaling with guardrails.** As policymakers consider broader models such as AHEAD, these results suggest that global budgets can coexist with stable margins while altering unit revenue patterns. Expansion strategies may need explicit guardrails and performance measures aligned with the intended mechanism—cost containment, access, and quality—rather than relying on margins as the primary indicator of success.^11,13^

Given the ongoing evolution of Maryland’s payment architecture and expansion pathways (TCOC; AHEAD), these findings motivate oversight mechanisms that explicitly track service mix and coding responses rather than relying on margins as the primary performance signal.^12,13^ Evidence from Maryland’s GBR context also indicates that broad prospective revenue models can generate spillover effects beyond spending and utilization targets, including increased centralization of selected complex surgical procedures at high-concentration hospitals after 2014.^22^ This pattern reinforces the need for complementary guardrails, monitoring service-line shifts and referral pathways, and assessing potential access and equity consequences when scaling global-budget approaches.

For federal policymakers considering broader expansion through TCOC- or AHEAD-like models, these results suggest that regulating the overall revenue ceiling alone may be insufficient; complementary oversight of coding, service intensity, and case-mix dynamics may also be necessary.

### 5.4. Limitations

Several limitations should be considered when interpreting these findings.

First, revenue per discharge is not a direct measure of negotiated or administered price. It reflects a composite of case mix, service intensity, payer mix, and accounting conventions. Accordingly, the findings are best interpreted as changes in revenue intensity per discharge rather than definitive price effects.^17^

Second, residual confounding remains possible. Although hospital and year fixed effects account for time-invariant hospital differences and common secular shocks, the two-state design cannot eliminate the influence of state-specific concurrent policies, market structure, payer dynamics, or consolidation trends.^15,16^ In addition, time-varying payer mix and case mix may have influenced the revenue-per-discharge outcome. Consistently harmonized measures of these covariates were not available with adequate completeness across the full matched panel and therefore were not included in the primary specification. In a restricted 2010–2019 sensitivity analysis using available case-mix and payer-mix measures, the revenue-per-discharge estimate was attenuated and no longer statistically significant, suggesting that changes in observable patient composition and payer structure may partly explain the main association.

Third, the analytic panel was unbalanced because hospital reporting and matched-file availability varied across fiscal years, and the volatility outcome additionally required consecutive-year observations by construction. This may affect representativeness if missingness was non-random. However, sensitivity analyses restricting the sample to hospitals observed in both the pre- and post-2014 periods yielded substantively similar results, suggesting that the main findings were not driven by panel composition across periods.

Fourth, the event-study and placebo analyses support the credibility of the design but do not eliminate uncertainty. Pre-policy coefficients were generally close to null, but several dynamic estimates were imprecise, and some post-policy coefficients varied across periods. The results therefore support cautious interpretation of average effects rather than precise claims about treatment dynamics or fine-grained heterogeneity over time.^14-16^

Fifth, the analysis is limited to hospital financial outcomes and does not directly evaluate utilization, quality, access, or distributional consequences. As a result, the study cannot determine whether the observed changes in revenue per discharge were associated with improved efficiency, changes in care patterns, or broader welfare effects.^3-5,8,11^

Finally, no formal a priori power analysis was conducted because the study used the full available matched hospital-year panel from the selected data sources and study period. Accordingly, the analysis may have been underpowered to detect small effects.

### 5.5. Future Research

Several extensions would strengthen the evidence base and address the limitations of the present study. First, future work should incorporate richer time-varying hospital characteristics and examine heterogeneity in effects across hospitals with different baseline margins, ownership structures, teaching status, system affiliation, and market positions.^16,20^ Second, comparator strategies could be expanded beyond Massachusetts by incorporating additional states or a multi-state donor pool, including synthetic control-style approaches, to reduce reliance on a single comparison market and strengthen causal interpretation.^14-16^ Third, further work is needed to decompose revenue per discharge into changes related to case mix, service intensity, coding, and accounting revenue shifts in order to clarify whether the post-2014 differential reflects compositional change or changes in unit revenue. Finally, linking hospital financial outcomes to utilization, quality, and access measures would help determine whether higher revenue per discharge under global budgets reflects efficiency gains, changes in care patterns, or unintended consequences for service delivery.^3-5,8,11^

## 6. Conclusion

Maryland’s all-payer global budget revenue (GBR) model, implemented in 2014, was associated with a statistically significant increase in inpatient revenue per discharge relative to Massachusetts, as measured in a matched hospital-year panel from 2010 to 2023. By contrast, there was no statistically detectable differential change in total margins after 2014 and no clear evidence of reduced year-to-year margin volatility during the COVID-19 period (2020-2022). Moreover, the revenue-per-discharge finding was sensitive to adjustment for available case-mix and payer-mix covariates in a restricted 2010–2019 sensitivity analysis, supporting a more cautious interpretation of that association. Taken together, these findings suggest that GBR may alter revenue intensity per inpatient episode without producing a corresponding shift in profitability or a measurable shock-buffering effect on margins. This pattern is consistent with a regulated revenue environment in which hospitals adjust service mix, coding, or intensity while overall financial performance remains broadly stable. The results, therefore, support a cautious interpretation of global budgets: they may change how hospitals generate revenue without necessarily improving profitability or guaranteeing financial stability during major disruptions. Policy discussions should treat financial stability as an empirical question and pair budget-based payment reforms with monitoring strategies that distinguish changes in revenue per discharge from underlying changes in case mix, intensity, and coding.

## Supplemental Material

Supplemental Material - Did Maryland’s All-Payer Global Budget Revenue Model Change Hospital Margins and Revenue per Discharge? A Difference-in-Differences Analysis of Maryland and Massachusetts, 2010–2023 Supplemental Material for Did Maryland’s All-Payer Global Budget Revenue Model Change Hospital Margins and Revenue per Discharge? A Difference-in-Differences Analysis of Maryland and Massachusetts, 2010–2023 by Kola Adegoke in Health Services Insights.

Supplemental Material - Did Maryland’s All-Payer Global Budget Revenue Model Change Hospital Margins and Revenue per Discharge? A Difference-in-Differences Analysis of Maryland and Massachusetts, 2010–2023 Supplemental Material for Did Maryland’s All-Payer Global Budget Revenue Model Change Hospital Margins and Revenue per Discharge? A Difference-in-Differences Analysis of Maryland and Massachusetts, 2010–2023 by Kola Adegoke in Health Services Insights.

## Data Availability

The Medicare hospital cost report panel used in this study was obtained from RAND (HCRIS-derived files) under a data-use and license agreement. Licensing restrictions prevent redistribution of the RAND data; qualified researchers may obtain access directly from RAND under the same terms. The CMS Provider of Services (POS) File—Hospital & Non-Hospital Facilities is publicly available from CMS and was accessed in this study as the quarterly extract Hospital_and_other.DATA.Q4_2025.csv. https://data.cms.gov/provider-characteristics/hospitals-and-other-facilities/provider-of-services-file-quality-improvement-and-evaluation-system Replication code and documentation sufficient to reproduce the analysis (conditional on obtaining access to the RAND data and downloading the public POS extract) are available via the Open Science Framework (OSF) associated project: https://osf.io/ybm3r and Registration: https://osf.io/qp4d5/overview.
