# Supplementary material for: Did Maryland’s All-Payer Global Budget Revenue Model Change Hospital Margins and Revenue per Discharge? A Difference-in-Differences Analysis of Maryland and Massachusetts, 2010–2023
Source: Health Serv Insights. 2026 Sep 4;19:11786329261486708. doi: 10.1177/11786329261486708 (PMC13547804; doi:10.1177/11786329261486708)
Supplement: Supplementary file 1 [file sj-pdf-1-his-10.1177_11786329261486708.pdf]

STROBE Checklist

Strengthening the Reporting of Observational Studies in Epidemiology was completed for the Maryland all-payer global budgets manuscript

Checklist table

Section/Topic	Item No.	STROBE recommendation	Reported on page no.	Author response / where addressed
**Title and abstract**	1	(a) Indicate the study's design with a commonly used term in the title or the abstract. (b) Provide in the abstract an informative and balanced summary of what was done and what was found.	Pg 1	The title and abstract identify the study as a comparative interrupted-panel analysis using two-way fixed-effects difference-in-differences. The abstract summarizes the background, matched hospital-year panel, data sources, outcomes, analytic approach, identification checks, principal estimates, and conclusions.
**Introduction**	2	Explain the scientific background and rationale for the investigation being reported.	Pg *1-3*	The introduction explains why hospital financial sustainability matters, why volume-dependent fee-for-service financing creates vulnerability, and why Maryland's all-payer global budgets are policy-relevant. It situates the study within prior work on spending, utilization, implementation, and COVID-era financial stress.
**Introduction**	3	State specific objectives, including any prespecified hypotheses.	Pg 3-6	The manuscript states three research questions and three corresponding hypotheses addressing total margin, log revenue per discharge, and COVID-period margin volatility.
**Methods**	4	Present key elements of study design early in the paper.	*Pg 6-8*	The Methods section states early that the study uses a comparative interrupted-panel design implemented as a two-way fixed-effects difference-in-differences analysis comparing Maryland with Massachusetts.
**Methods**	5	Describe the setting, locations, and relevant dates, including periods of recruitment, exposure, follow-up, and data collection.	*Pg 6-8*	The study setting is hospital-year financial performance in Maryland and Massachusetts. The analysis period spans fiscal years 2010-2023, with Maryland's 2014 global budget implementation defining the primary policy break and 2020-2022 defining the COVID-period stress-test window.
**Methods**	6	(a) Give the eligibility criteria, and the sources and methods of selection of participants. Describe methods of follow-up. (b) For matched studies, give matching criteria and number of exposed	*Pg 6-8*	Eligible observations were hospital-year records in the merged RAND HCRIS and CMS POS files for Maryland and Massachusetts retained after a 1:1 matched-only merge by CMS Certification Number and

Section/Topic	Item No.	STROBE recommendation	Reported on page no.	Author response / where addressed
		and unexposed.		fiscal year. The panel is unbalanced because reporting varies across hospitals and years. This is not an individually matched exposed/unexposed cohort study, so matching criteria for persons are not applicable.
**Methods**	7	Clearly define all outcomes, exposures, predictors, potential confounders, and effect modifiers. Give diagnostic criteria, if applicable.	*Pg 6-8*	The manuscript defines the exposure as Maryland's post-2014 all-payer global budget period relative to Massachusetts. Outcomes are total margin, log inpatient revenue per discharge, and absolute year-over-year change in total margin during COVID. The main interaction terms, event-study structure, placebo timing, and selected provider characteristics from the POS file are described.
**Methods**	8*	For each variable of interest, give sources of data and details of methods of assessment (measurement). Describe comparability of assessment methods if there is more than one group.	*Pg 6-8*	Hospital financial variables were obtained from RAND HCRIS-derived files and provider characteristics from the CMS Provider of Services file. Measures were constructed consistently across Maryland and Massachusetts using CMS Certification Number and fiscal year. Variable definitions for total margin, log revenue per discharge, and volatility are described in the Outcomes and Variable Definitions subsection.
**Methods**	9	Describe any efforts to address potential sources of bias.	*Pg 8*	Potential bias was addressed through hospital and year fixed effects, hospital-clustered standard errors, event-study diagnostics for pre-trends, a placebo falsification test using a false 2012 intervention date, and sensitivity analyses using winsorized volatility outcomes. The manuscript also discusses limits related to residual confounding and the two-state design.
**Methods**	10	Explain how the study size was arrived at.	*Pg 6-8*	The analytic sample reflects all matched hospital-year observations available after merging the RAND HCRIS and CMS POS files for Maryland and Massachusetts over 2010-2023. If no a priori power analysis was performed, the Discussion notes this as a limitation.
**Methods**	11	Explain how quantitative variables were handled in the analyses. If applicable, describe which groupings were chosen and why.	*Pg 6-8*	Quantitative outcomes were analyzed at the hospital-year level. Total margin was modeled in levels, inpatient revenue per discharge was log-transformed to reflect unit revenue intensity, and volatility was defined as the absolute year-over-year change in total margin for consecutive fiscal years. Post-policy and COVID-period indicators were prespecified based on policy timing and the pandemic window.

Section/Topic	Item No.	STROBE recommendation	Reported on page no.	Author response / where addressed
**Methods**	12	(a) Describe all statistical methods, including those used to control for confounding. (b) Describe any methods used to examine subgroups and interactions. (c) Explain how missing data were addressed. (d) If applicable, explain how loss to follow-up was addressed. (e) Describe any sensitivity analyses.	*Pg 6*	Statistical methods are described in detail: two-way fixed-effects DiD models with hospital and year fixed effects and hospital-clustered standard errors; an event-study specification; a placebo test; and COVID-period volatility models using a Maryland × COVID interaction. Missingness is handled by estimating each outcome model on the maximal non-missing sample for that outcome, with outcome-specific Ns reported. Sensitivity analyses include winsorized volatility outcomes. Because the study uses administrative hospital-year panel data rather than follow-up of individual participants, loss to follow-up is not applicable in the conventional sense.
**Results**	13*	(a) Report numbers of individuals at each stage of study. (b) Give reasons for non-participation at each stage. (c) Consider use of a flow diagram.	*Pg 9-13*	The Results report the number of matched hospital-year observations overall and by state, as well as outcome-specific non-missing counts. The Methods explain that the panel is unbalanced because of variation in hospital reporting across years. A formal participant flow diagram is not included because the study uses administrative hospital-year records rather than individual enrollment stages.
**Results**	14*	(a) Give characteristics of study participants and information on exposures and potential confounders. (b) Indicate number of participants with missing data for each variable of interest. (c) Summarize follow-up time.	*Pg 9-13*	Descriptive results report the number of hospital-years contributed by each state and the number of non-missing observations for each outcome. Time-varying provider characteristics are described as data sources in Methods; additional descriptive provider characteristics can be added if included in the final tables. Follow-up time corresponds to the hospital-year panel across 2010-2023.
**Results**	15*	Report numbers of outcome events or summary measures over time.	*Pg 9-13*	The manuscript reports summary measures over time through descriptive tables and state-year trend figures for total margin and log revenue per discharge, as well as regression estimates for the main outcomes and volatility models.
**Results**	16	(a) Give unadjusted estimates and, if applicable, confounder-adjusted estimates and their precision. Make clear which confounders were adjusted for and why they were included. (b) Report category boundaries when continuous variables were categorized. (c) If relevant, consider translating estimates of relative risk into absolute risk for a meaningful time period.	*Pg 9-13*	The Results provide estimated coefficients, clustered standard errors, p-values, and sample sizes for the main DiD models, the volatility models, and the placebo test. The manuscript makes clear that hospital and year fixed effects are included in the primary models. For log revenue per discharge, the coefficient is translated into an approximate percent change to aid interpretation.
**Results**	17	Report other analyses done, eg analyses of subgroups and	*Pg 9-13*	Other analyses reported include the event-study diagnostic, placebo

Section/Topic	Item No.	STROBE recommendation	Reported on page no.	Author response / where addressed
		interactions, and sensitivity analyses.		falsification test, and winsorized volatility sensitivity analyses. The Discussion interprets these results cautiously and as supportive of identification rather than as separate primary findings.
**Discussion**	18	Summarize key results with reference to study objectives.	*Pg 13-14*	The Discussion opens with a concise summary of the three principal findings and links them directly to the corresponding hypotheses and research questions.
**Discussion**	19	Discuss limitations of the study, taking into account sources of potential bias or imprecision. Discuss both direction and magnitude of any potential bias.	*Pg 14-15*	The Limitations subsection addresses outcome validity, residual confounding in a two-state design, unbalanced panel structure and missingness, uncertainty around parallel trends and dynamic estimates, and the restricted scope of financial outcomes. The discussion is framed cautiously and avoids overstatement.
**Discussion**	20	Give a cautious overall interpretation of results considering objectives, limitations, multiplicity of analyses, results from similar studies, and other relevant evidence.	*Pg 13-15*	The Discussion provides a cautious interpretation of higher revenue per discharge without detectable margin or volatility effects, situates the findings relative to prior studies of Maryland's model and pandemic-era financial stress, and emphasizes what can and cannot be concluded from the design.
**Discussion**	21	Discuss the generalisability (external validity) of the study results.	*Pg 13-15*	The manuscript discusses generalisability in relation to Maryland's distinctive all-payer global budget system, the Massachusetts comparator, and the broader relevance of prospective payment reform for initiatives such as AHEAD while noting that results may not generalize fully to other states or payment environments.
**Other information**	22	Give the source of funding and the role of the funders for the present study and, if applicable, for the original study on which the present article is based.	___________	The manuscript stated the funding situation and conflict-of-interest disclosures in the journal front matter. Data sources are acknowledged, and lack any funding

** STROBE items 8, 13, 14, and 15 have different wording across cohort, case-control, and cross-sectional versions. This checklist uses cohort-style wording because the manuscript is an observational hospital-year panel study.*
