# Supplementary material for: Did Maryland’s All-Payer Global Budget Revenue Model Change Hospital Margins and Revenue per Discharge? A Difference-in-Differences Analysis of Maryland and Massachusetts, 2010–2023
Source: Health Serv Insights. 2026 Sep 4;19:11786329261486708. doi: 10.1177/11786329261486708 (PMC13547804; doi:10.1177/11786329261486708)
Supplement: Supplementary file 2 [file sj-pdf-2-his-10.1177_11786329261486708.pdf]

Appendix Table S1. Outcome missingness by state and fiscal year, 2010–2023

State	Fiscal year	N hospital-years	% missing total margin	% missing margin volatility
MA	2010	108	12.96	100.00
MA	2011	109	13.76	13.76
MA	2012	109	13.76	13.76
MA	2013	108	12.96	12.96
MA	2014	107	13.08	13.08
MA	2015	102	12.75	12.75
MA	2016	101	11.88	12.87
MA	2017	100	11.00	13.00
MA	2018	97	10.31	11.34
MA	2019	98	11.22	12.24
MA	2020	98	11.22	12.24
MA	2021	99	10.10	12.12
MA	2022	99	10.10	10.10
MA	2023	96	8.33	8.33
MD	2010	61	14.75	100.00
MD	2011	60	11.67	13.33
MD	2012	60	13.33	13.33
MD	2013	59	11.86	16.95
MD	2014	58	10.34	12.07
MD	2015	59	10.17	11.86
MD	2016	60	10.00	11.67
MD	2017	60	10.00	10.00
MD	2018	60	10.00	10.00
MD	2019	59	10.17	10.17
MD	2020	58	12.07	12.07
MD	2021	59	13.56	15.25
MD	2022	59	13.56	13.56
MD	2023	58	13.79	13.79

Notes: Percent missing total margin indicates the share of hospital-year observations in the merged analytic panel with missing values for the total-margin outcome. Percent missing margin volatility indicates the share missing the volatility outcome, which is additionally undefined in the first available year for each hospital and requires consecutive-year observations by construction.
